# Supplementary material for: Origin of Heterogeneous Stripping of Lithium in Liquid Electrolytes
Source: ACS Nano. 2023 May 31;17(11):10218–28. doi: 10.1021/acsnano.3c00329 (PMC10278170; doi:10.1021/acsnano.3c00329)
Supplement: Supplementary file 1 — nn3c00329_si_001.pdf [file nn3c00329_si_001.pdf]

Supporting Information
Origin of heterogeneous stripping of lithium in liquid electrolytes

Martin Werres[1,2], Yaobin Xu[4], Hao Jia[5], Chongmin Wang[4], Wu Xu[5], Arnulf Latz[1,2,3], and Birger Horstmann *[1,2,3]

[1]Institute of Engineering Thermodynamics, German Aerospace Center (DLR), Wilhelm-Runge-Str. 10, 89081 Ulm, Germany
[2]Helmholtz Institute Ulm (HIU), Helmholtzstr. 11, 89081 Ulm, Germany
[3]Department of Electrochemistry, University of Ulm, Albert-Einstein-Allee 47, 89081 Ulm, Germany
[4]Environmental Molecular Sciences Laboratory, Pacific Northwest National Laboratory, Richland, WA, 99354, USA
[5]Energy and Environment Directorate, Pacific Northwest National Laboratory, Richland, WA, 99354, USA

1 Implementation & Mathematical Details

We assume the dissolution to be reaction-limited, *i.e.*, the diffusion of lithium in the electrolyte to be sufficiently fast. Further we assume a constant concentration of Li^+ at the surface which is valid for stripping current densities before diffusion limitations occur:

$$j < j_{\mathrm{diff}} = F \cdot D \cdot \frac{\Delta c}{L} \approx 300\,\mathrm{A\,m^{-2}}, \tag{SI-1}$$

where $F = 96\,485\,\mathrm{C/mol}$ is the Faraday constant, $D = 3 \cdot 10^{-10}\ m^2/s$ is the typical diffusion constant of a $LiPF_6$ electrolyte[1], $\Delta c = 1\% \cdot 1\,\mathrm{mol/l}$ is a small change in the Li^+ concentration in the typical length scale of a whisker $L = 1\,\mu\mathrm{m}$. This is a conservative estimate, as the ions diffuse in the bulk electrolyte and not along the whisker. Therefore the length scale is smaller and consequently the reaction limited current larger, undermining that diffusion limitations do not play a role in the dissolution process for small current densities.

*Corresponding Author: birger.horstmann@dlr.de

As the initial geometry, we assume a cone-like shape with a spherical tip, similar to the structures observed in experiments, see **Figure 1**. This ansatz allows us to use a cylindrical symmetry. We connect the cone and the sphere via a smeared out Heaviside function.

$$r_0(z) = (1 - \Theta(z)) \cdot R + \Theta(z) \cdot \sqrt{(aR)^2 - (z - L - b)^2} \tag{SI-2}$$

with

$$\Theta(z) = \frac{1}{2} \tanh \frac{(z - L) \cdot c}{R} + 0.5 \tag{SI-3}$$

where $R = 100\,\text{nm}$ is the whisker radius, $L = 5\,\mu\text{m}$ is the whisker cone length, $a = 1.1$ indicates that the sphere on top of the whisker has a slightly larger radius than the whisker cone, $b = 0.1R$, and $c = 8$ determines the width of the smeared out Heaviside function.

Our model starts with the geometrical assumption of the whisker in Equation SI-2. We evaluate the function in steps of $\Delta z = 15\,\text{nm}$ for $z < 0.8L$ and $\Delta z = 3\,\text{nm}$ for $z >= 0.8L$. The points are used as the initial whisker surface and the SEI. The SEI points are assumed to be rigid, *i.e.*, they do not change during dissolution. In reality, the SEI shell falls together, due to a negative pressure beneath the SEI surface. This is not important for our simulation of the whisker dissolution, as this happens after the Li-SEI bond is broken. The whisker surface moves according to the equations of motion, Equations 4 & 5:

$$\frac{\partial r(\xi)}{\partial t} = \frac{\dot{z}}{\sqrt{\dot{r}^2 + \dot{z}^2}} \cdot \frac{V_\text{M}}{F} \cdot J(\xi)$$
$$\frac{\partial z(\xi)}{\partial t} = \frac{-\dot{r}}{\sqrt{\dot{r}^2 + \dot{z}^2}} \cdot \frac{V_\text{M}}{F} \cdot J(\xi)$$

The geometrical parts $\dot{z}/\sqrt{\dot{r}^2 + \dot{z}^2}$ and $-\dot{r}/\sqrt{\dot{r}^2 + \dot{z}^2}$ assure that the movement is perpendicular to the surface. The local dissolution current density $J(\xi)$ determines the dissolution velocity, given by Eq. 6:

$$J_\text{BV}(\xi) = J_0 \left[e^{\frac{-F\Delta\Phi}{2RT}} - e^{\frac{\mu(\xi)}{RT}} e^{\frac{F\Delta\Phi}{2RT}} \right],$$

For calculating the Butler Volmer rate, we need to determine the potential step $\Delta\Phi = \Phi - \Phi_0$ relative to the lithium metal and the chemical potential μ. The potential step $\Delta\Phi$ is determined by solving the Galvanostatic condition:

$$I = \int_{A_0} f J_0 \text{d}A = \int J \text{d}A = \int J(\xi) 2\pi r \sqrt{\dot{r}^2 + \dot{z}^2} \text{d}\xi \tag{SI-4}$$

where f is the fraction of the initial current density to the effective exchange current density.

The Butler-Volmer equation can be modified using the simple formula for Marcus-Hush-Chidsey kinetics by Bazant and co-workers[2]:

$$
\begin{aligned}
J_{\mathrm{MHC}}(\xi) =& J_0 \cdot A \cdot e^{\frac{\mu(\xi)}{2RT}} \cdot \sqrt{\pi\lambda} \cdot \tanh(\eta/2) \\
&\cdot \operatorname{erfc}\left(\frac{\lambda - \sqrt{1+\sqrt{\lambda}+\eta^2}}{2\sqrt{\lambda}}\right),
\end{aligned}
\tag{SI-5}
$$

where $A = J_{\mathrm{BV}}(\eta = 0)/J_{\mathrm{MHC}}(\eta = 0)$ makes sure that the current descriptions give the same value at zero overpotential, $\lambda = 10$ is the dimensionless reorganization energy and $\eta = \eta(\xi) = (\mu + \Delta\Phi)/2RT$ is the local overpotential.

First, the chemical potential needs to be determined by evaluating Equation 8. From Equation 7 we get

$$
\begin{aligned}
\int g \mathrm{d}z &= \int \sigma(d,\alpha) 2\pi r \sqrt{1+r'^2} \mathrm{d}z \\
\Leftrightarrow g &= \sigma(d,\alpha) 2\pi r \sqrt{1+r'^2}
\end{aligned}
\tag{SI-6}
$$

The interfacial tension $\sigma(d,\alpha)$ depends on the distance d, which can be calculated by:

$$
d = \operatorname{sign}(r - r_0)\sqrt{(r-r_0)^2 + (z-z_0)^2} \tag{SI-7}
$$

and the angle α, where

$$
\cos\alpha = \frac{\dot{z}\dot{z}_0 + \dot{r}\dot{r}_0}{\sqrt{(\dot{r}^2+\dot{z}^2)(\dot{r}_0^2+\dot{z}_0^2)}}. \tag{SI-8}
$$

according to Equation 9, where we use

$$
\sigma_{\|}(d) = \sigma_{\mathrm{Li}} \begin{cases} -1 + 6\left(\frac{d}{a}\right)^4 + 16\left(\frac{d}{a}\right)^3 + 12\left(\frac{d}{a}\right)^2 & d > -a \\ 1 & d \leq -a \end{cases} \tag{SI-9}
$$

to interpolate between a state where lithium is adhered to the SEI and is surrounded by liquid electrolyte, and

$$
f(\alpha) = \cos 2\alpha \tag{SI-10}
$$

mainly for numerical reasons. For evaluating Equation 7, we need to determine $\partial g/\partial r$ and $\partial g/\partial r'$. This requires to explicitly calculate all the partial derivatives.

$$
\begin{aligned}
\frac{\partial g}{\partial r} =& \frac{\partial \sigma(d,\alpha)}{\partial d}\frac{\partial d}{\partial r} 2\pi r\sqrt{1+r'^2} \\
&+ \frac{\partial \sigma(d,\alpha)}{\partial \cos\alpha}\frac{\partial \cos\alpha}{\partial r} 2\pi r\sqrt{1+r'^2} \\
&+ \sigma(d,\alpha) 2\pi\sqrt{1+r'^2} \\
=& \frac{\partial \sigma(d,\alpha)}{\partial d}\frac{\partial d}{\partial r} 2\pi r\sqrt{1+r'^2} \\
&+ \frac{\partial \sigma(d,\alpha)}{\partial \cos\alpha}\frac{\partial \cos\alpha}{\partial r'_0} r''_0 \frac{\partial z_0}{\partial r} 2\pi r\sqrt{1+r'^2} \\
&+ \sigma(d,\alpha) 2\pi\sqrt{1+r'^2}
\end{aligned}
\tag{SI-11}
$$

$$\begin{aligned}\frac{\partial g}{\partial r'} =&\frac{\partial \sigma(d,\alpha)}{\partial \cos\alpha}\frac{\partial \cos\alpha}{\partial r'}2\pi r\sqrt{1+r'^2}\\&+\sigma(d,\alpha)2\pi r\frac{r'}{\sqrt{1+r'^2}}\end{aligned} \tag{SI-12}$$

For calculating $\partial z_0/\partial r$ we make use, that we evaluate the SEI at the point closest to the whisker surface. Thus, the orthogonality relation

$$r_0' \cdot \left(\frac{r_0 - r}{z_0 - z}\right) = -1 \tag{SI-13}$$

holds. With this we can determine the derivatives implicitly:

$$\begin{aligned}\frac{\partial z_0}{\partial r} &= \frac{\partial\left[z - r_0'(r_0 - r)\right]}{\partial r}\\&= r_0' - r_0''(r_0 - r)\frac{\partial z_0}{\partial r} - r_0'^2\frac{\partial z_0}{\partial r}\\&= \frac{r_0'}{(1 + r_0''(r_0 - r) + r_0'^2)}\end{aligned} \tag{SI-14}$$

With this, we can finally evaluate Equation 7:

$$\begin{aligned}\mu =&V_\mathrm{M}\sigma_\perp\left(\frac{1}{R_1}+\frac{1}{R_2}\right)\\&+V_\mathrm{M}(\sigma_\parallel-\sigma_\perp)\left(f(\alpha)-\frac{\partial f(\alpha)}{\partial\cos\alpha}\cos\alpha\right)\left(\frac{1}{R_1}+\frac{1}{R_2}\right)\\&+V_\mathrm{M}(\sigma_\parallel-\sigma_\perp)\frac{\partial f(\alpha)}{\partial\cos\alpha}\left(\frac{1}{d+R_1^0}+\frac{1}{d+R_2^0}\right)\\&+V_\mathrm{M}\frac{\partial\sigma_\parallel}{\partial d}\left(f(\alpha)\cos\alpha+\sin^2\alpha\frac{\partial f(\alpha)}{\partial\cos\alpha}\right)\\&+V_\mathrm{M}(\sigma_\parallel-\sigma_\perp)\frac{\partial^2 f(\alpha)}{\partial(\cos\alpha)^2}\sin^2\alpha\left(\frac{1}{R_2}-\frac{\cos\alpha}{d+R_2^0}\right).\end{aligned} \tag{SI-15}$$

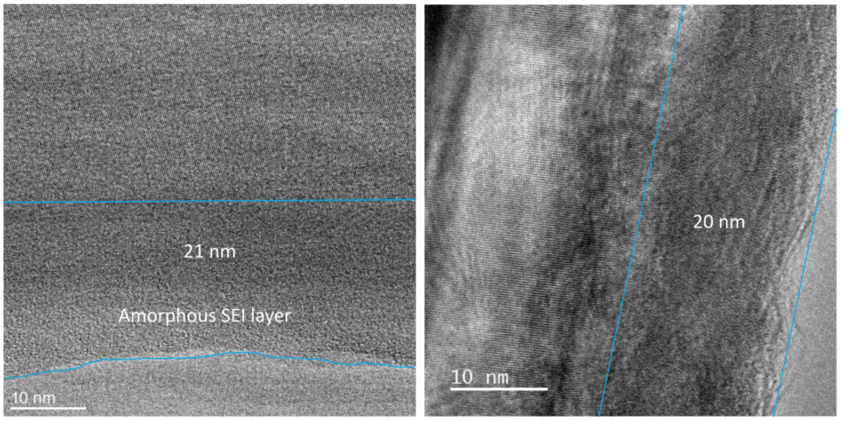

(a) Cryo TEM image of the SEI after plating at $1\,\mathrm{Am^{-2}}$.

(b) Cryo TEM image of the SEI after plating at $10\,\mathrm{Am^{-2}}$.

Figure SI-1: Cryo TEM observations of SEI thickness after plating at different current densities.

2 Additional experimental details and results

2.1 SEI thickness

In order to investigate the SEI thickness, we performed high resolution cryo TEM, see Fig. SI-1.For both plating current densities of $1\,\mathrm{Am^{-2}}$ and $10\,\mathrm{Am^{-2}}$, the SEI is identified to be amorphous with no clear nanostructure and around $20\,\mathrm{nm}$ in thickness.

2.2 Plating and stripping behavior at higher current density

For probing higher current densities, we build a coin cells with the same properties as described in the Methods Section. For the plating/stripping cycling, we applied a plating current of $10\,\mathrm{Am^{-2}}$ for 10 mins and then discharged the cell with $-10\,\mathrm{Am^{-2}}$ until a cut-off voltage of $1\,\mathrm{V}$. The plated capacity equals the one in the experiment at lower current density.

In the following we show the observed Li morphology and SEI structure and composition at higher current density.

To investigate the chemical compostion of the whisker and its covering SEI, we performed HAADF STEM with EDS and EELS mapping. The results are shown in Fig. SI-2. The SEI is rich in O and C with little amount of F. There is no striking difference in the SEI composition compared to whiskers formed at lower current density; see Fig. 1.

After stripping, we investigate the remaining structure on the Cu grid. We observe that only hollowed-out SEI shells remain that cannot be dissolved; see

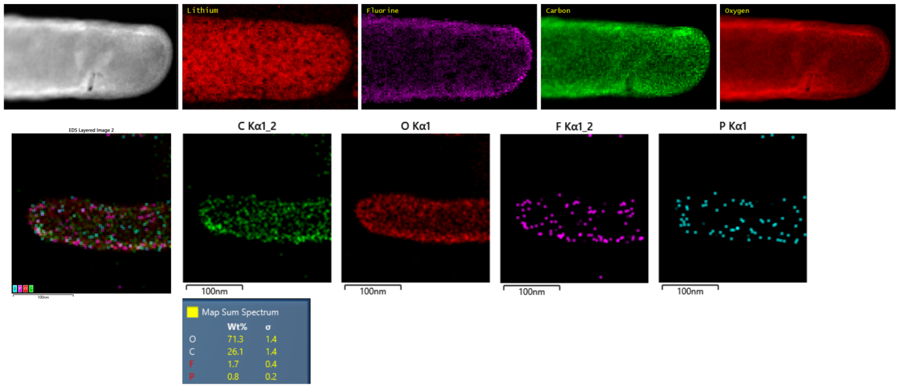

Figure SI-2: High-angle annular dark field (HAADF) scanning transmission electron microscopy image of the whisker and its tip with the corresponding electron energy loss spectroscopy (EELS) elemental mapping for a plating current density of $10\,\mathrm{A m^{-2}}$.

Fig. SI-3. Note, that in Fig. SI-3 (a) the “blobbs” are ice crystals seeding on the SEI. In Fig. SI-3 (b) it can be seen that during the stripping, the SEI can break.

While after plating, the Cu grid is completely covered by whiskers, after stripping, the whiskers are mostly dissolved; see Fig. SI-4.

3 Additional simulation results

3.1 Local current distibution

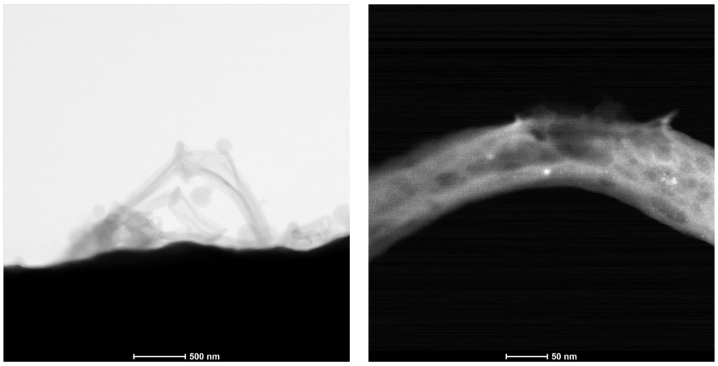

Figure SI-3: Cryo TEM image of lithium whiskers after stripping. Only empty SEI shells remains.

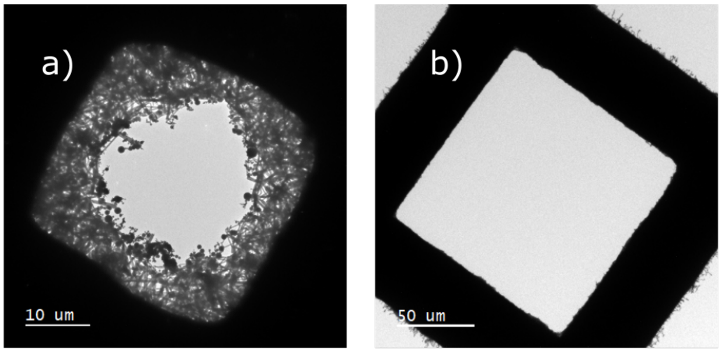

Figure SI-4: Cryo TEM image of the copper grid (a) after 10 minutes plating at $10\,\mathrm{Am^{-2}}$ and (b) after stripping at $10\,\mathrm{Am^{-2}}$ until the cut-off voltage is reached.

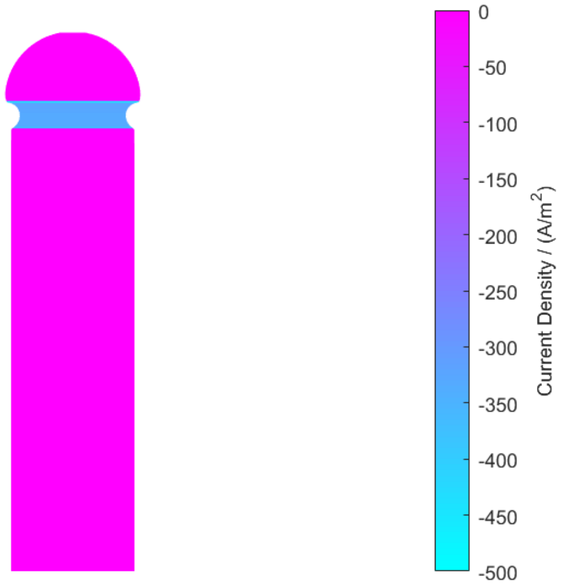

Figure SI-5: Local current distribution for the scenario of stripping at $0.1\,\mathrm{mAcm^{-2}}$ during the formation of a droplet.

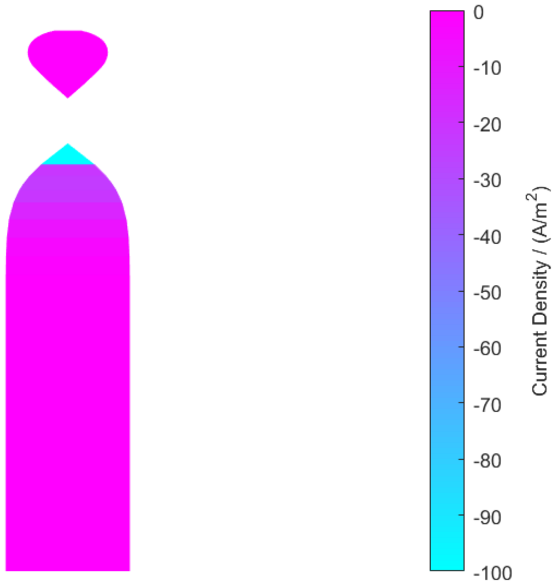

Figure SI-6: Local current distribution for the scenario of stripping at $0.1\,\mathrm{mAcm^{-2}}$ after the droplet formation.

References

[1] Valoen LO, Reimers JN. Transport Properties of $LiPF_6$-Based Li-Ion Battery Electrolytes. Journal of The Electrochemical Society. 2005;152(5):A882. Available from: https://doi.org/10.1149/1.1872737.

[2] Zeng Y, Smith RB, Bai P, Bazant MZ. Simple formula for Marcus–Hush–Chidsey kinetics. Journal of Electroanalytical Chemistry. 2014 Dec;735:77-83. Available from: https://doi.org/10.1016/j.jelechem.2014.09.038.
